# Corrigendum to “Intestinal, portal, and peripheral profiles of daikenchuto (TU‐100)'s active ingredients after oral administration”

**DOI:** 10.1002/prp2.1206

**Published:** 2024-05-16

Watanabe J., et al. *Pharmacol Res Perspect. 2015; 3: e00165*.

Several errors have been identified in Table 4. The correct table is shown below:

**TABLE 4:** Recovery of ingested ingredients found in the human ileal fluid.

Herbs	Japanese pepper			Ginseng	
Ingredients	HAS	HBS	GS	Rb1	Rg1
Amout contained in ingested TU‐100 (ng/5 g TU‐100)	2800000	505000	204000	685000	685000
β‐glucuronidase treatment	−	−	−	−	−
Amount (ng/ileum)					
Min	B.L.Q	B.L.Q	B.L.Q	B.L.Q	B.L.Q
Max	10700	6810	139000	531000	788000
Median	B.L.Q	B.L.Q	8380	405000	627000
% of ingested TU‐100*					
Max	0.4	1.3	68.1	77.5	115.0
Median	B.L.Q	B.L.Q	4.1	59.1	91.5

Herbs	Ginger							
Ingredients	6G		10G		6S		10S	
Amout contained in ingested TU‐100 (ng/5 g TU‐100)	558000		162000		760000		197500	
β‐glucuronidase treatment	−	+	−	+	−	+	−	+
Amount (ng/ileum)								
Min	B.L.Q	B.L.Q	B.L.Q	B.L.Q	B.L.Q	B.L.Q	B.L.Q	B.L.Q
Max	3870	76100	2040	11600	1250	5710	5020	6970
Median	B.L.Q	41200	B.L.Q	7630	B.L.Q	3000	B.L.Q	1550
% of ingested TU‐100*								
Max	0.7	13.6	1.3	7.2	0.2	0.8	2.5	3.5
Median	B.L.Q	7.4	B.L.Q	4.7	B.L.Q	0.4	B.L.Q	0.8

* Value represents (the amount of each ingredient in the ileal contents)/(the amount of ingredients contained in ingested TU‐100) × 100.

We apologize for this error.

